# Bilateral anomalous veins arising from the popliteal vein with variant termination of the short saphenous vein: a case report

**DOI:** 10.1590/1677-5449.202501452

**Published:** 2026-03-23

**Authors:** Hisham Muhsen, Naveen Kumar, Abdalla Ahmed Eldaw Elamin, Vijay Paul Samuel, Rana Aly Mohamed Elbeshbeishy

**Affiliations:** 1 RAK Medical and Health Sciences University – RAKMHSU, RAK College of Medical Sciences, Ras Al Khaimah, United Arab Emirates.

**Keywords:** short saphenous vein, anomalous vein, Giacomini’s vein, vascular anatomy, venous variation, veia safena parva, veia anômala, veia de Giacomini, anatomia vascular, variação venosa

## Abstract

This report explores the presence of bilateral anomalous veins with variant termination of the small saphenous veins (SSV) in both lower limbs. The SSV originated normally on the right side, draining into an anomalous vein from the popliteal vein. This vein then ascended cranially to the back of the thigh and terminated in the thigh veins. On the left side, the SSV had its normal draining pattern into the popliteal vein. However, slightly distal to the saphenopopliteal junction, an anomalous vein originated from the popliteal vein, which ascended upwards to the back of the thigh and terminated in the thigh veins. Presence of variant veins are likely to complicate venous ultrasonography and make procedures more difficult for sonographers to perform. Prior knowledge of variant patterns of superficial veins of the lower limbs is useful for clinicians during coronary bypass procedures, as these vessels are commonly used in such surgeries.

## Resumo

Este relato explora a presença de veias anômalas bilaterais associadas a terminação variante das veias safenas parvas (VSP) em ambos os membros. No lado direito, a VSP apresentou origem habitual, drenando para uma veia anômala proveniente da veia poplítea. Essa veia, por sua vez, ascendeu cranialmente pela região posterior da coxa, terminando nas veias da coxa. No lado esquerdo, a VSP manteve seu padrão usual de drenagem na veia poplítea. Entretanto, um pouco distalmente à junção safeno-poplítea, uma veia anômala originou-se da veia poplítea, ascendendo pela região posterior da coxa e terminando nas veias da coxa. A presença de veias variantes pode dificultar a ultrassonografia venosa e tornar a realização de procedimentos mais complexa para os sonografistas. O conhecimento prévio sobre padrões variantes das veias superficiais do membro inferior é útil para clínicos durante procedimentos de *bypass* coronário, já que esses vasos são frequentemente utilizados nessas cirurgias.

## INTRODUCTION

The short saphenous vein (SSV) begins as a continuation of the lateral marginal vein behind the lateral malleolus. It ascends along the lateral side of the calcaneal tendon in the lower third of the calf, then curves medially and pierces the deep fascia of the calf at the midline. It then ascends along the gastrocnemius and emerges between the deep fascia and gastrocnemius, usually under the lower limit of the popliteal fossa. As it continues up, it passes between the heads of the gastrocnemius muscle and terminates in the popliteal vein which lies 3-7.5 cm above the knee joint, within the popliteal fossa.^1^

Variations in SSV termination and other superficial veins are not uncommon and are widely documented in the scientific literature. One of particular relevance is the thigh extension of the SSV referred to as “Giacomini’s vein” (GV), first described by Carlo Giacomini in 1873. He published his work under the title “Anatomical observations to serve the study of the venous circulation of the lower limbs,” in which he detailed numerous patterns of the SSV extending, terminating, or connecting to different veins in the thigh.^2^

The SSV can receive reflux from Giacomini’s vein through the perineal veins, neighboring tributary veins, and perforators of the thigh.^3^ Duplex ultrasonography is the gold standard method for detecting variations like these and precisely diagnosing venous disease in the lower limb. It is noninvasive, fast, and simple to perform, guiding management by accurately describing the extent of disease.^4^ Identifying variant veins that cause incompetence is essential for proper treatment because varicose vein surgery is regularly performed.^5^ Numerous ways of categorizing variations in lower limb veins have been proposed, the most relevant to this case being the Giacomini classification, which was later refined by Oliveira et al.^6^,^7^ (Table 1).

**Table 1:** The de Oliveira^7^ classification of SSV based on variant drainage patterns.

**Type**	**SSV drainage pattern**	**Sub type & drainage pattern**
Type 1	Drains into PV	a. Directly to PV
		b. Bifurcates and one division drains into PV and another into GSV
Type 2	Femoral vein/veins of posterior compartment of thigh/into GSV	c. To deep veins of the thigh
		d. Divides and drains into deep veins of the thigh and to GSV
		e. To GSV
Type 3	Into veins of the leg (without reaching PV)	f. Communicates with GSV (at leg region)
		g. Veins of gastrocnemius

SSV = short saphenous vein; PV = popliteal vein; GSV = great saphenous vein.

This case report discusses a clinical insight into the existence of bilateral anomalous veins with variant termination of the SSV. Cases like these contribute to existing anatomical knowledge of the lower limb, emphasizing the importance of awareness of variant drainage and the existence of anomalous veins prior to procedures like preoperative ultrasound scanning and autologous grafting.

This report is derived from a formalin fixed cadaver which was donated for medical education use. This study adheres to the principles of the Helsinki declaration and complies with local ethical guidelines. We (the authors) confirm that all necessary ethical approvals and consent for the use of the cadaver were obtained. No identifiable patient data was used in this report.

## CASE REPORT

**Figure 1:**
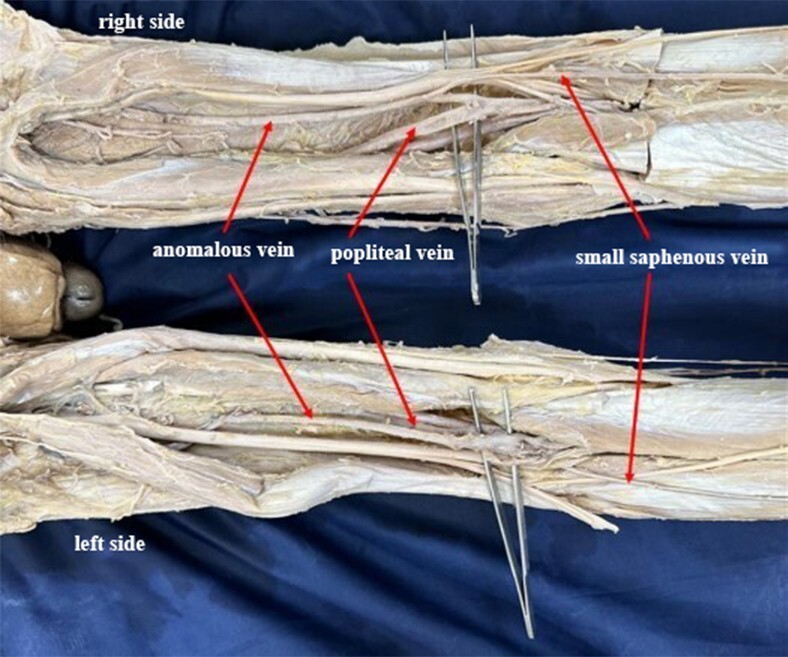
Shows the termination of the small saphenous veins and anomalous veins at the popliteal fossa of both sides.

**Figure 2:**
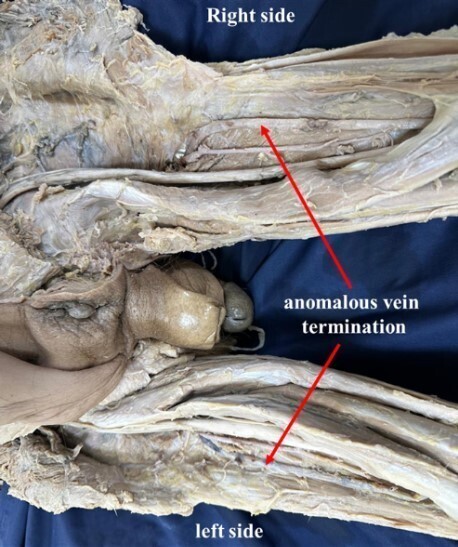
Shows the termination of the bilateral anomalous veins into the veins of the thigh on both sides.

**Figure 3:**
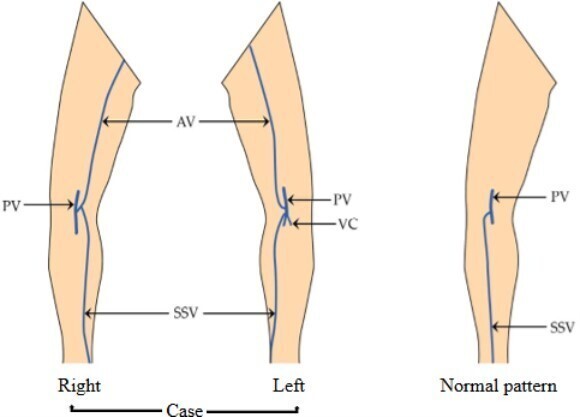
Schematic representation of bilateral venous variations at lower limbs. AV = Anomalous vein; PV = Popliteal vein; SSV = Short saphenous vein; VC = Venae comitantes of the tibial artery.

During a routine cadaveric dissection of the popliteal fossa, for MD students of a medical fraternity, we observed anomalous veins bilaterally. The origin of the SSV was normal in both limbs. On the right side, it drained into an anomalous vein that commenced from the popliteal vein. This anomalous vein further ascended cranially to the back of the thigh and eventually terminated into the veins of the back of the thigh. On the left side, the SSV had its normal draining pattern into the popliteal vein. However, slightly distal to the saphenopopliteal junction, an anomalous vein originated from the popliteal vein, which in its further course, ascended upwards to the back of the thigh and terminated in the veins of the thigh (Figures 1 and 2). Furthermore, on the left side, the small saphenous vein drained into the popliteal vein at the site where the popliteal vein was formed by the venae comitantes of the tibial arteries (Figure 3). No other vascular variations were observed.

## DISCUSSION

Reports discussing the prevalence of Giacomini’s vein in the literature have been widely inconsistent, with incidence rates fluctuating according to study design. More specifically, cadaveric studies have reported the highest prevalence, reaching up to 95%; ultrasound assessments have shown considerable variability, ranging from 2% to 86%; and one phlebographic study reported a rate as low as 2.5%.^3^ What makes this report exceptionally unique is the presence of bilateral anomalous veins, as most reports only discuss unilateral variations.

Embryologically, the cervical and lumbar intersegmental vessels begin to develop the anastomosing channels as they progress towards their respective extremities during vasculogenesis.^8^ Pre-axial (ventral) and post-axial (dorsal) veins, which later develop into the great and short saphenous veins in the lower limbs, respectively, drain the venous blood to the heart via the cardinal venous system.^8^,^9^

Morphological differences in lower limb veins are not uncommon and do not always pose health risks; however, it is necessary to be aware of them prior to certain procedures. It is particularly important to highlight that the SSV can receive reflux from GV, allowing varicose veins to form in the posterior leg. A recent study published in 2024 by Engelhorn et al.^3^ found the incidence of GV to be 14%, with 29.4% of these veins having reflux. In addition, almost a fourth of GVs caused reflux into the SSV, demonstrating that preoperative scanning is crucial for surgical planning. Such circumstances, paired with cosmetic complaints and risk of thrombosis formation, can indicate the need for surgical intervention after identifying the reflux pattern.^3^,^10^ From a clinical standpoint, these interventions need to consider both the variant form of SSV along with any anomalous morphology, failing to do so could contribute to recurrent varicose veins.^6^,^11^

A similar pattern of bilateral venous anomalies on the posterior aspect of both thighs was reported by Cardoso et al.^12^ They reported that the left vein originated from the popliteal vein and drained into the deep femoral vein, while the right vein originated from veins in the anterior tibial region and drained into the internal iliac vein. Both veins ascended cranially alongside the sciatic nerve as persistent sciatic veins and passed through the adductor magnus muscle to reach their point of drainage.^12^

Scanning for anatomical variations is crucial for surgical planning, especially when the great saphenous vein is unavailable for grafting. The SSV is considered a suitable option for saphenous vein grafts but is limited by its shorter length. Bilateral existence of anomalous veins in the lower limb, particularly when continuous with the short saphenous vein (similar to the right lower limb in this case), could possibly serve as a solution, offering additional length for use as a bypass conduit in infrainguinal venous reconstruction.^13^

In normal circumstances, where the great saphenous vein is used as a coronary artery bypass graft, vascular surgeons need to take note of any variations and communications with the SSV, reducing the chances of varicose vein recurrence.^14^ However, special care needs to be taken prior to utilizing these veins to make sure that the quality of the saphenous vein is not jeopardized, reducing the likelihood of graft failure.

## CONCLUSION

Variations in the veins of lower extremities, such as GV (variant form of SSV), are prone to reflux and are likely to complicate venous ultrasonography, making procedures more difficult for sonographers to perform. Recognizing patterns of such veins is crucial for clinicians and surgeons alike in vascular surgeries and varicose vein treatment, in which brushing off anomalies may lead to misdiagnosis, recurrence, and procedural complications. Acknowledging such variants enhances the accuracy of diagnostic imaging, guiding safe surgical interventions involving the superficial lower limb veins.

## Data Availability

Data sharing does not apply to this article, as no data were generated or analyzed.
